# Evaluating Older Adults’ Engagement and Usability With AI-Driven Interventions: Randomized Pilot Study

**DOI:** 10.2196/64763

**Published:** 2025-01-24

**Authors:** Marcia Shade, Changmin Yan, Valerie K Jones, Julie Boron

**Affiliations:** 1 College of Nursing, University of Nebraska Medical Center, 985330 Nebraska Medical Center, Omaha, NE, 68198, United States, 1 4025596641; 2 College of Journalism and Mass Communications, University of Nebraska Lincoln, Lincoln, NE, United States; 3 Department of Gerontology, University of Nebraska at Omaha, Omaha, NE, United States

**Keywords:** voice assistant, interventions, usability, engagement, personality, older adults, aging, technology, artificial intelligence, AI, self-management, pilot trial, chronic, musculoskeletal pain, AI assistant, Alexa, user experience, digital health, digital intervention, mobile phone

## Abstract

**Background:**

Technologies that serve as assistants are growing more popular for entertainment and aiding in daily tasks. Artificial intelligence (AI) in these technologies could also be helpful to deliver interventions that assist older adults with symptoms or self-management. Personality traits may play a role in how older adults engage with AI technologies. To ensure the best intervention delivery, we must understand older adults’ engagement with and usability of AI-driven technologies.

**Objective:**

This study aimed to describe how older adults engaged with routines facilitated by a conversational AI assistant.

**Methods:**

A randomized pilot trial was conducted for 12-weeks in adults aged 60 years or older, self-reported living alone, and having chronic musculoskeletal pain. Participants (N=50) were randomly assigned to 1 of 2 intervention groups (standard vs enhanced) to engage with routines delivered by the AI assistant Alexa (Amazon). Participants were encouraged to interact with prescribed routines twice daily (morning and evening) and as needed. Data were collected and analyzed on routine engagement characteristics and perceived usability of the AI assistant. An analysis of the participants’ personality traits was conducted to describe how personality may impact engagement and usability of AI technologies as interventions.

**Results:**

The participants had a mean age of 79 years, with moderate to high levels of comfort and trust in technology, and were predominately White (48/50, 96%) and women (44/50, 88%). In both intervention groups, morning routines (n=62, 74%) were initiated more frequently than evening routines (n=52, 62%; *z*=−2.81, *P*=.005). Older adult participants in the enhanced group self-reported routine usability as good (mean 74.50, SD 11.90), and those in the standard group reported lower but acceptable usability scores (mean 66.29, SD 6.94). Higher extraversion personality trait scores predicted higher rates of routine initiation throughout the whole day and morning in both groups (standard day: B=0.47, *P*=.004; enhanced day: B=0.44, *P*=.045; standard morning: B=0.50, *P*=.03; enhanced morning: B=0.53, *P*=.02). Higher agreeableness (standard: B=0.50, *P*=.02; enhanced B=0.46, *P*=.002) and higher conscientiousness (standard: B=0.33, *P*=.04; enhanced: B=0.38, *P*=.006) personality trait scores predicted better usability scores in both groups.

**Conclusions:**

he prescribed interactive routines delivered by an AI assistant were feasible to use as interventions with older adults. Engagement and usability by older adults may be influenced by personality traits such as extraversion, agreeableness, and conscientiousness. While integrating AI-driven interventions into health care, it is important to consider these factors to promote positive outcomes.

## Introduction

Digital technologies may be beneficial for helping older adults stay connected and directing them to health resources [1]. Newer, technology-based interventions may provide a way to assist with the self-management of chronic illnesses and improve social connections for older adults [2]. Traditional technologies such as computers and mobile tablets have been adopted by aging adults but can be abandoned depending on acceptance and ease of use [3]. Newer technologies such as conversational voice assistants are a type of artificial intelligence (AI) that have the capacity to assist and have basic interactions with individuals. Older adults can engage with conversational AI through voice, which offers an advantage over spending extensive time learning a new program or manually operating small sized device features. Among Americans aged 18 years and older, 62% use an AI conversational voice assistant on devices such as smart speakers, smartphones, TV remotes, in-car systems, computers, and tablets among others [4]. With the increasing population of older adults, conversational voice assistants may be an optimal modality to deliver interventions.

The use of conversational voice assistants has been studied as an intervention for chronic conditions such as heart failure, lung disorders, and mental health [5]. Their use has been explored for promoting behaviors such as physical activity and for health care support [6,7]. Studies are emerging in relation to conversational voice assistants independently influencing loneliness, social isolation, and pain [8-13]. These studies have primarily focused on acceptability, barriers, and the design of conversational voice assistants for older adult populations. In addition, a recent review of the literature only focused on the use and acceptability of conversational voice assistants to reduce loneliness; none of the conversational voice assistant-based interventions were personalized [13,14]. Kocabelli et al [15] found several conversational voice assistants delivered interventions that were personalized, but lacked theoretical framework or evidence-based approach for implementation. Further exploration is needed to understand older adults’ engagement with conversational voice assistants to ensure optimal outcomes when used as a modality for intervention delivery.

An individual’s personality can influence acceptance and needs to be considered as a part of usability and the engagement with technology-driven interventions [16]. In younger populations, openness to experience and agreeableness, have been shown to be positively related to usability of technology [17].

Personality traits are characteristics of individuals that typically remain stable over the adult life course. Adults from their mid-to-late 20s and older were found to score similarly across the lifespan on the Big Five traits (ie, openness to experience, conscientiousness, extraversion, agreeableness, and neuroticism [18]. The OCEAN acronym is used to describe these traits. The “O” personality trait of being open to experience is the appreciation of a variety of experiences, “C” conscientiousness is the exhibition of self-discipline, and “E” extraversion is the amount of engagement one has with the external world. The trait of “A” is, agreeableness, which is a concern for social harmony and the “N” is neuroticism trait, which is the tendency of a person to exhibit negative emotions. An individual’s personality traits play a role in health care, particularly when implementing patient-centered care and encouraging intervention adherence [19]. Loneliness, which is prevalent in older adults, may influence the presentation of personality traits. A meta-analysis documented negative associations between loneliness and extraversion, agreeableness, conscientiousness, and openness, and a positive association between loneliness and neuroticism [20]. Another study found that loneliness was associated with varying levels of extraversion, neuroticism, and agreeableness [21]. When using technology to deliver interventions in older adults, engagement may differ in those that self-report loneliness; however, this warrants further exploration.

An individual’s personality may influence the engagement and user experience with AI technology. Within the realm of traditional technologies, the strongest correlates of older adults’ perceived usefulness and ease of use of computers were the personality traits of agreeableness and openness [22,23]. Openness to experience has been associated with increased probability of internet use [16]. Furthermore, higher agreeableness and lower neuroticism in older adults have been associated with perceived usability with an automated vehicle [24].

Since the AI boom, the relationship between the personality traits of older adults and use of conversational voice assistants has not been extensively explored and may impact engagement with AI driven interventions. A commercially available AI voice assistant, that is, Alexa, was used to deliver prescribed interactive routines as nonpharmacological interventions for pain and loneliness. In this analysis of the data, the following research questions were explored:

1. When do older adults initiate the various prescribed routines?
2. What are the older adults’ perceived usability scores with different intervention routines delivered by AI through conversational voice assistants?
3. What is the relationship between older adults’ personality and their engagement with the different intervention routines?
4. What is the relationship between older adults’ personality and the perceived usability of different intervention routines delivered by AI through conversational voice assistants?

## Methods

### Overview

This study was a 12-week pilot randomized trial conducted with a convenience sample of participants who were 60 years of age or older, self-reported living alone, spoke English, and experienced pain symptoms. A sample size calculation was performed a priori for this pilot study. A sample of 59 participants was calculated to account for 10% study attrition, 80% power, 0.35 effect size, and 0.05 significance. The Mini Cog was used to screen individuals to ensure cognitive ability of participants [25]. Individuals with memory loss or with previous or current use of conversational voice assistants were excluded. Informed consent was obtained prior to initiating study related activities. Once participants were enrolled, they were given a study identification number, and a computerized sorter randomly assigned them to one of the two intervention groups. Participants were compensated for their time during this study. One $25 dollar gift card was given to participants after completing baseline questionnaires, one $25 gift card after completing 12-week questionnaires, and were allowed to keep the Amazon Echo Dot device.

### Intervention Groups

Prescribed routines were used for the intervention, such as the frequency of prescription medications (eg, daily, twice daily, as needed). The routines were created based on nonpharmacologic strategies [26] and selected from evidence-based interventions that have a positive influence on pain and loneliness [2,27,28]. After screening and enrollment, participants were randomly assigned to intervention groups.

The 2 intervention groups included the conversational voice assistant standard (CVA-S) routine and the conversational voice assistant enhanced (CVA-E) routine. Standard routines were more generalized and static, while the enhanced routines were tailored, as it has been well documented that individualized or tailored interventions increase engagement and adherence [29-31]. The AI routines in the CVA-S group included a generalized (without personalization) Alexa greeting such as stating, “good morning” or “good evening.” The routines consisted of a combination of a generalized (1) meditation, (2) game, (3) music, and (4) jokes. The CVA-S group’s “as needed” routine was a general greeting and interactive activity. The AI routines in the CVA-E group included more sociable greetings that were personalized to each participant’s name preference such as stating, “hi there, Jim” or “good evening, Betty.” The CVA-E routine consisted of a combination of personalized (1) pain location and pain type meditation (2) interactive game preferences, (3) genre or musical artist, and (4) choice of preferred joke style, devotionals, interactive stretching and or other interactive activities.

### Routine Programming

The conversational voice assistant “Alexa” was used for this study because the 65+ population prefers Alexa among commercially available voice assistants [32]. Routines were programmed within the Alexa app using available actions and skills created by software app developers. Actions are standard preprogrammed interactions such as playing music or telling a joke, and skills are voice-activated apps such as health fitness, education, games, podcasts, and meditations that can be used with Alexa and the smart speaker. Before skill selection for this study, research personnel reviewed ratings and tested the skills to make sure they worked and would be appropriate for older populations. Routines were initiated by participants speaking to an Amazon Echo Dot device and stating “Alexa, start my routine,” which then cued the sequence of selected actions and skills.

### Measures and Instruments

Data collected from participants was de-identified and included characteristics such as demographics, health history, and comfort with and trust of technology, assessed on a 4-point scale ranging from 1 (none) to 4 (high). The Big Five Inventory (BFI) was used to measure personality traits of the participants. The BFI contains 44 questions that represent the different traits of openness (Cronbach *α*=0.77), conscientiousness (Cronbach *α*=0.72), extraversion (Cronbach *α*=0.79), agreeableness (Cronbach *α*=0.78), and neuroticism (Cronbach *α*=0.70) [18,33].

Feasibility outcomes included capturing engagement with the AI assistant routines and self-reported usability. Engagement with routines was measured at the end of the study. Participants’ voice assistant profiles were securely accessed to track the date and time stamps on the incidence of routine initiation with the standard and enhanced loneliness routines. Usability was measured using the System Usability Scale (SUS) administered at the end of the study. A 10-item system usability scale (ranging from 0 to 100) was created (Cronbach α=0.75) [34].

### Data Analysis

The data analysis strategy for this study was multifaceted, aimed at understanding the impact of different intervention conditions on routine initiation rates, the influence of personality traits on engagement with Alexa-based routines, and perceptions of system usability. Given the nature of the data and the research questions, the analysis used both descriptive statistics and inferential statistical tests to explore these relationships and test hypotheses. This comprehensive data analysis strategy, incorporating both nonparametric tests for hypothesis testing and regression analysis for exploring relationships between personality traits and key outcomes, facilitated a nuanced understanding of the factors influencing routine initiation and system usability in the context of Alexa-based interventions.

Descriptive statistics in addition to normality tests were conducted. Initial analysis involved descriptive statistics (mean, median, SD, skewness, and kurtosis) to summarize the central tendency and dispersion of routine initiation rates and system usability scores. The Shapiro-Wilk test was used to assess the normality of distributions for these variables. Given the significant results from the Shapiro-Wilk test indicating nonnormal distributions for both routine initiation rates and system usability scores, nonparametric tests were chosen for hypothesis testing.

Nonparametric tests were applied to test the differences in routine initiation rates and SUS scores across conditions (morning vs evening and standard vs enhanced). The Wilcoxon Signed-Rank test was used to compare morning and evening routine initiation rates within participants. Mann-Whitney *U* test was used to test differences between the standard and enhanced conditions in terms of routine initiation rates and SUS scores. This test was chosen due to its suitability for comparing 2 independent samples without assuming normal distribution.

### Personality Traits Analysis

Multiple regression analyses were conducted to explore the relationship between participants’ personality traits and their engagement with Alexa routines, as well as the perceived usability of the system. These analyses were performed for the total sample and separately for each condition (standard vs enhanced) to determine if personality traits differentially influenced outcomes based on the intervention type. The choice of multiple regression was guided by the interest in assessing the predictive power of multiple independent variables (personality traits) on a continuous dependent variable (number of routine initiations or system usability scores).

### Ethical Considerations

The University of Nebraska Medical Center Institutional Review Board approved the study protocol (IRB #0177‐21-EP).

## Results

### Demographic findings

The study included 50 participants with mean age 79.24 (SD 7.85; median 78, range 65‐98). The majority (44/50, 88%) were female (male: 6/50, 12%) and most (48/50, 96%) participants identified as White (African American: 2/50, 44%). Most of the older adults were either widowed (22/50, 44%) or legally divorced or separated (21/50, 42%), while the remaining participants were either living apart from their partner due to health reasons ( 5/50, 10%) or single (2/50, 4%). Most ((40/50, 80%) participants had completed a college degree or higher, while 16% (8/50) had completed high school, and (2/50, 4%) had attended trade school. The majority (32/50, 64%) reported incomes between US $10,000-$50,000, while 4% (2/50) earned below US $10,000, 14% (7/50) earned between US $50,001-$90,000, and 18% (9/50) earned above US $90,000. Participants reported moderate to high levels of comfort (mean 2.75, SD 0.67) and trust in technology (mean 2.94, SD 0.91) on a scale of 1-5.

### Engagement and Perceived Usability of Prescribed Routines

Participants were encouraged to engage with and initiate the routines twice daily for 12 weeks, which equated to 168 interactions in total. As shown in Table 1, the rates of routine initiations were significantly higher among participants in the CVA-E group than the CVA-S group. While observing the prescribed time of day for engagement, the routine initiation rates were higher in the morning.

**Table 1.** Comparisons of daily routine initiations of participants in the CVA-S^a^ and CVA-E^b^ groups in this pilot study of older adults’ engagement with 12-week artificial intelligence (AI)-driven interventions.

Condition	Routine initiation (Time of day)	Routine initiation frequency (actual number of interactions/total number of possible interactions, %)	Number of initiations, mean (SD)	*z* score	*P* value
Full sample (n=50)	Morning	62/84^c^, 74	62.13 (32.85)	−2.81	.005
Full sample (n=50)	Evening	52/84, 62	51.94 (32.13)		
CVA-E (n=26)	Entire day	126/168^d^, 75	126.53 (59.9)	−1.98	.048
CVA-S (n=24)	Entire day	101/168, 60	100.56 (54.88)		
CVA-E (n=26)	Morning	62/84, 74	62.24 (31.86)	−3.16	.002
CVA-S (n=24)	Morning	49/84, 58	49.01 (29.17)		
CVA-E (n=26)	Evening	44/84, 52	52.29 (32.3)	−0.13	.90
CVA-S (n=24)	Evening	44/84, 52	51.55 (32.63)		

a CVA-S: conversational voice assistant standard.

b CVA-E: conversational voice assistant enhanced.

c Number of interactions during either morning and evening.

d Total number of interactions during the day.

Given the variances in rates of routine initiations, and a notable preference for morning over evening initiations across conditions, group comparisons were conducted using Wilcoxon analyses to examine the interaction between intervention timing (morning vs evening) and intervention groups (CVA-S vs CVA-E using the Mann-Whitney *U* test). As presented in Table 1, the CVA-E group initiated “morning routines” significantly more often than those in the CVA-S group. In the evening, the rates of routine initiation were not significantly different between the groups.

The perceived usability of the AI assistant showed favorable results. The SUS scores of the overall sample of participants were “good” (mean 70.56, SD 10.58). When conditions were analyzed separately, the CVA-E group’s SUS scores were good (mean 74.50, SD 11.90), while the CVA-S group scores were “OK” (mean 66.29, SD 6.94).

### Personality and Engagement With Prescribed Routines

**Figure 1.**
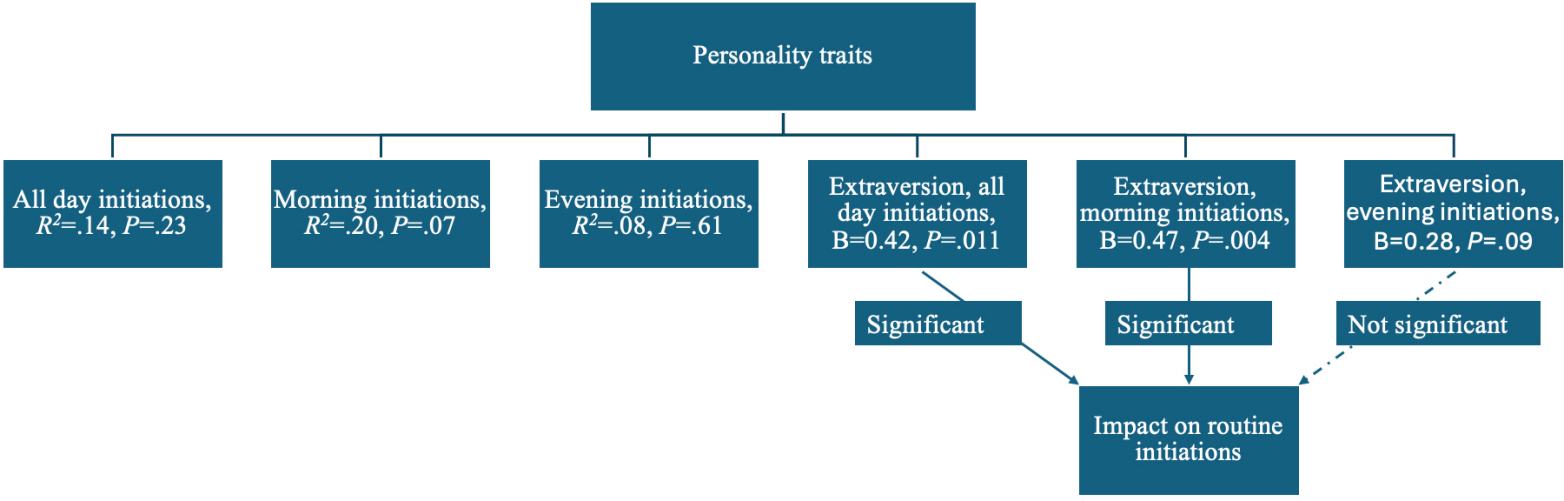
Personality trait predictors of routine initiations throughout the day in this pilot study of older adults’ engagement with 12-week artificial intelligence (AI)-driven interventions.

As displayed in Figures1  2, the overall model including all Big Five personality traits was not a good fit for predicting of the daily number of routine initiations (*F*_5,44_=1.45, *P*=.23, *R*^*2*^=.14), morning routine initiation (*F*_5,44_=2.19, *P*=.07, *R*^*2*^=.20), or evening routine initiation (*F*_5,44_=0.72, *P*=.61, *R^2^*=.08) at α=.05.

However, the extraversion personality trait was significantly associated with routine initiations. It was a significant predictor of overall daily routine initiations (B=0.42, *t*=2.65, *P*=.01) and initiation of morning routines (B=0.47, *t*=3.07, *P*=.004), but not the evening routines (B=0.28, *t*=1.73, *P*=.09). The other 4 personality traits did not predict routine initiations.

**Figure 2.**
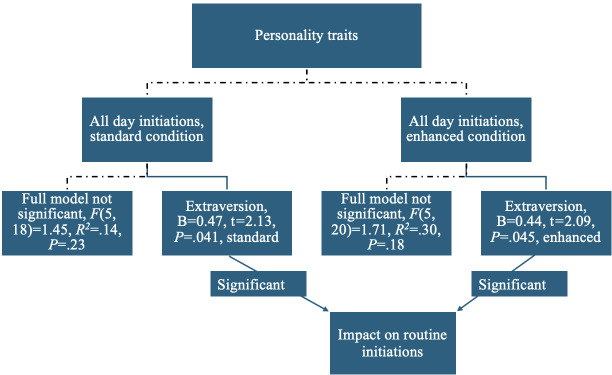
Personality trait predictors of daily routine initiations of participants in the CVA-S (conversational voice assistant standard) and CVA-E (conversational voice assistant enhanced) groups in this pilot study of older adults’ engagement with 12-week artificial intelligence (AI)-driven interventions.

**Figure 3.**
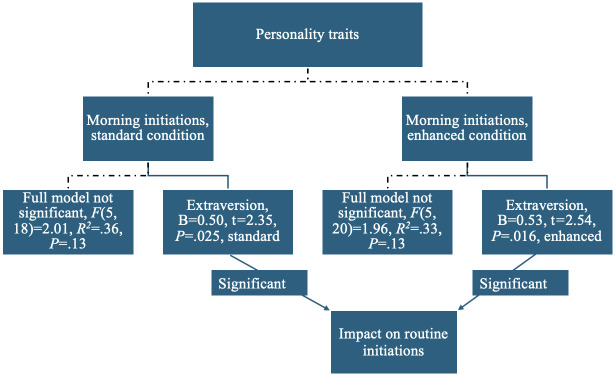
Personality trait predictors of morning routine initiations of participants in the CVA-S (conversational voice assistant standard) and CVA-E (conversational voice assistant enhanced) groups in this pilot study of older adults’ engagement with 12-week artificial intelligence (AI)-driven interventions.

Similar patterns were observed when comparing the CVA-S and CVA-E groups. Extraversion significantly predicted all routine initiations in the CVA-S group, (B=0.47, *t*=2.13, *P*=.041 and the CVA-E group, B=0.44, *t*=2.09, *P*=.045). As displayed in Figure 3, extraversion was a significant predictor of morning routine initiations in the CVA-S group, B=0.50, *t*=2.35, *P*=.03, and the CVA-E group, B=0.53, *t*=2.54, *P*=.02. None of the Big Five personality traits significantly predicted evening routine initiations in either group.

### Personality and Perceived Usability of Prescribed Routines

**Figure 4.**
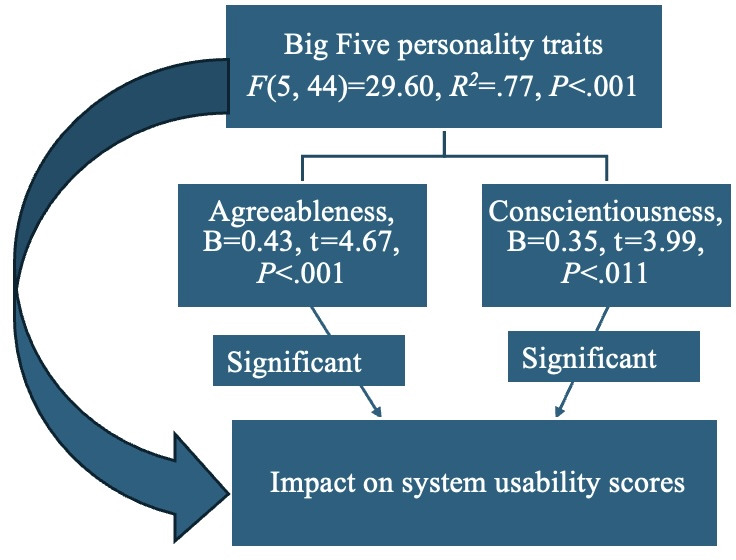
Personality trait predictors of participant self-reported usability in this pilot study of older adults’ engagement with 12-week artificial intelligence (AI)-driven interventions.

As depicted in Figure 4, among the Big Five personality traits, only agreeableness (B=0.43, *t*=4.67, *P*<.001) and conscientiousness (B=0.35, *t*=3.99, *P*<.001) positively predicted SUS scores in the overall sample (*F*_5,44_=29.60, *P*<.001, *R*^2^=0.77).

**Figure 5.**
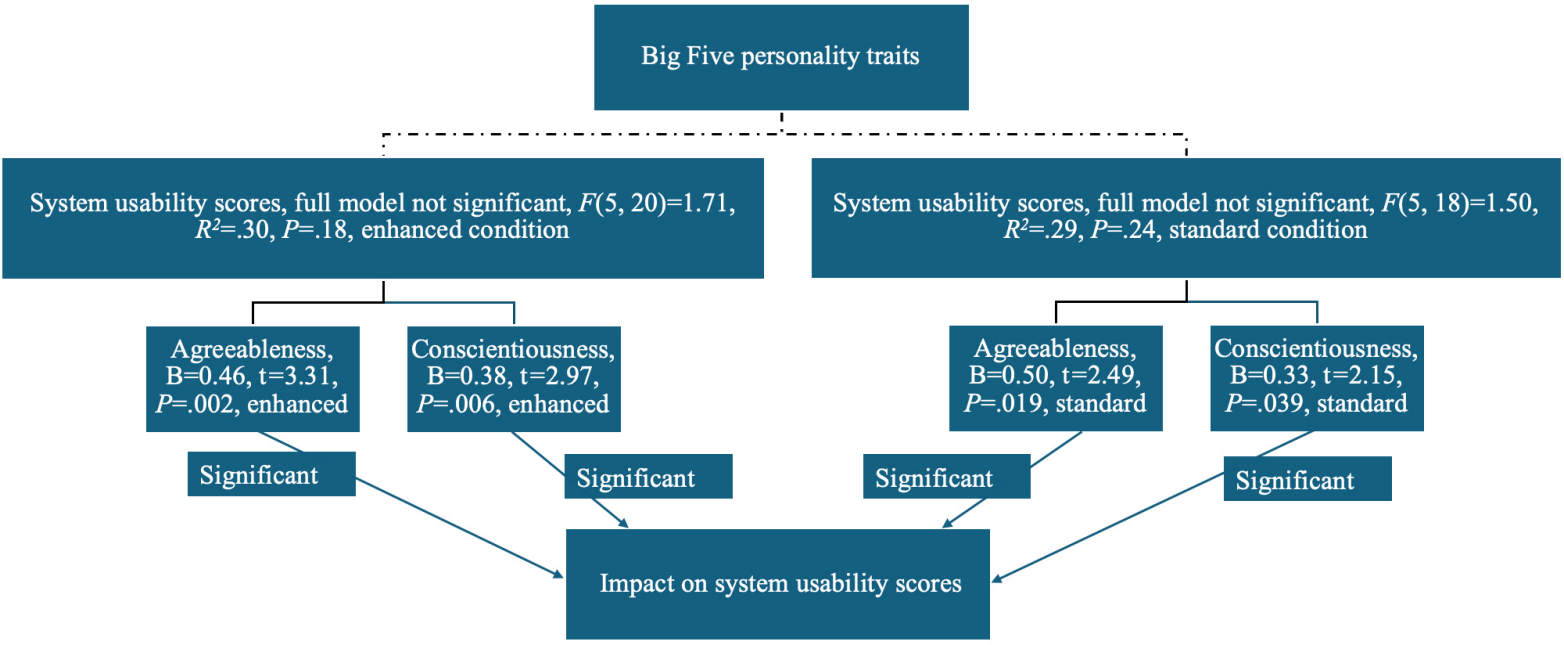
Personality trait predictors of participants self-reported usability in the CVA-S (conversational voice assistant standard) and CVA-E (conversational voice assistant enhanced) groups in this pilot study of older adults’ engagement with 12-week artificial intelligence (AI)-driven interventions.

As shown in Figure 5, when analyzed separately, both the CVA-S (B=0.50, *t*=2.49, *P*=.02) and CVA-E (B=0.46, *t*=3.31, *P*=.002) groups reported agreeableness respectively and conscientiousness (B=0.33, *t*=2.15, *P*=.04; B=0.38, *t*=2.97, *P*=.006 respectively) were positively associated with SUS scores.

## Discussion

### Principal Findings

Technology is becoming a popular vehicle for delivering interventions for health care and self-management. In this analysis, we examined older adults’ engagement with and usability of AI-delivered routine interventions. Overall, the older participants engaged with the assistant throughout the 12 weeks of the study. Perceived usability was acceptable, with routine initiation rates being higher in the morning. Personality traits of the participants in this study may have impacted engagement with the prescribed routines. The following discussion provides additional insights into the findings.

### Older Adults’ Engagement With Prescribed Routines and Perceived Usability

Our study revealed significant differences in routine initiation rates between morning and evening sessions, with participants demonstrating a greater propensity to engage in prescribed routines in the morning. A potential explanation is behavioral habit. Engagement and adherence may be greater in the morning than in the evening because of internal behavioral cues [35]. Another potential explanation for this finding lies within circadian rhythm science. Circadian rhythms are internal processes that regulate the sleep-wake cycle, repeating roughly every 24 hours. They influence physical, mental, and behavioral changes in living organisms. Circadian rhythms are crucial for determining human sleep patterns and have significant implications for designing technology that aligns with these natural processes [36]. Research suggests that morning hours are associated with higher alertness and cognitive functioning, which could facilitate the initiation of health-related routines [37]. The enhanced intervention components may have amplified these effects, indicating that tailored interventions can effectively increase engagement with health-promoting behaviors. The integration of technology with human circadian rhythms or habits may enhance intervention engagement and adherence.

Another possible explanation for the higher initiation rates in the morning could be arthritic pain. Inclusion criteria was self-reported pain symptoms and most of the participants reported a diagnosis of arthritis. Commonly, individuals experience stiffness in the morning that may impact pain symptoms and need for management [38]. In older adults with musculoskeletal pain, technology-based routines or interventions may be best encouraged in the morning.

### Older Adults’ Personality and Engagement

The results of this study highlight the influence of personality traits on engagement and usability. Extraversion was a significant predictor of routine initiation, particularly in the morning, which is consistent with previous research indicating that extraverted individuals are more likely to engage in health-promoting activities [39]. Extraverts, characterized by their energy, enthusiasm, and sociability, might have the most inherent motivation for social interaction and stimulation. When they perceived the routines as opportunities for engaging experiences or personal growth, it may have made the process of initiating routines more appealing [40,41]. The finding that this effect was more pronounced in the morning suggests that, consistent with their circadian rhythm, extraverts may be prone to engage with technology during early hours, setting a positive tone for the rest of their day.

Consistent with Kortum and Oswald [17], agreeableness was one personality trait that aligned with usability in our sample of older adults. Agreeable and conscientious personality predicted higher perceived usability, suggesting that these traits may influence individuals’ perceptions and interactions with technology-based health interventions. It is worth noting the older participants in this study reported moderate to high levels of comfort and trust in technology. Agreeable individuals, known for their cooperative and trusting nature, may be more predisposed to view the Alexa-based intervention in a positive light, attributing any potential usability issues to their own learning curve rather than the system’s design flaws. This optimistic viewpoint may lead to higher perceived usability. Conscientious individuals, with their focus on diligence, organization, and reliability, may appreciate systems that are consistent and efficient. The participants’ positive evaluation of the system’s usability might stem from a recognition of its potential to support their goal-oriented behavior and routine adherence. The structured nature of the preset routines could align well with their preference for orderly environments, further enhancing their perception of the system’s usability.

The study reveals a nuanced differentiation between participants’ engagement with prescribed routines and their perceptions of system usability, emphasizing the distinct roles of personality traits. Extraversion was found to drive the frequency of routine initiations, particularly in the morning, suggesting that an action-oriented engagement is influenced by a desire for stimulation and interaction. Older adults with extraverted personality traits may be more likely to seek that interaction and treat AI with anthropomorphic properties [42]. Anthropomorphism is when a human attributes human traits to entities or devices that are not human [43]. Researchers have found that people interact with conversational voice assistants in anthropomorphic ways, greeting them, asking them personal questions, exhibiting polite behaviors such as saying “please” and “thank you,” and reacting to what the voice assistant did or did not say [12]. Thus, there is a possibility that anthropomorphic interactions with the conversational voice assistants may have impacted engagement and usability of conversational voice assistants to deliver interventions.

Conversely, agreeableness and conscientiousness were associated with more favorable perceptions of system usability, indicating a cognitive evaluation process that values trust, adaptability, and reliability. This distinction underscores the importance of differentiating the motivational drivers behind the use of technology-based health interventions and the cognitive evaluations that shape user satisfaction. By acknowledging the interplay between action-oriented engagement and cognitive evaluations, designers can create more effective, user-friendly interventions tailored to diverse user preferences and personality profiles.

Extraverts, who are stimulated by social interaction and are more action-oriented, particularly in the morning, may benefit from interventions scheduled during this time. Health platforms can incorporate adaptive scheduling features that customize the timing of prompts based on the user’s personality profile, thus optimizing the likelihood of engagement and adherence which could impact health outcomes.

Furthermore, understanding the role of personality traits in the acceptance and usability of health technologies can inform the development of more personalized, effective, and user-friendly interventions. The significant role of agreeableness and conscientiousness in shaping perceptions of system usability suggests that these traits should be considered when designing user interfaces. Users high in agreeableness and conscientiousness value trust, adaptability, and reliability. Therefore, health interventions should prioritize these design elements to cater to their cognitive evaluation processes. For example, features that provide consistent feedback, allow for personalization, and are easy to navigate could be more appealing to these users. Researchers have found that conversational voice assistants exhibit “personality type” traits. The Google and Alexa AI assistants have personality traits of functional intelligence, sincerity, sociability, and creativity [44]. The personality and voice of the assistant has shown to enhance engagement [45]. Future interventions could be tailored for optimal engagement when matching AI assistants and human dominant personalities.

Although the results of this pilot study are informative there are limitations worth noting. There were an even number of participants in each intervention group, however, our sample was relatively small and lacked diversity. The findings are not generalizable to all older adults who report loneliness and pain. Our participants self-reported loneliness and pain, but our study may not have enrolled individuals who were experiencing more intense loneliness and pain. Primary care providers could help us identify older adults with more severe pain and or loneliness. Those without regular internet access in their home due to location (eg, rural areas) or lack of broadband access (due to personal choice or financial restraints) would not be able to interact with conversational voice assistants. Some of these sources of variation could also interact with personality differences. For example, those that choose not to have internet in their homes for personal reasons, may differ from those that do. Furthermore, exploring how to engage those with differing personality characteristics is an important area for future research, so that routines are individualized enough to produce an impact on daily life. Finally, our study was 12-weeks in duration; a longer study would be beneficial to explore the length of time needed to integrate routines that impact well-being and to sustain long term impact.

### Conclusions and Implications

Commercially available conversational voice assistants have the potential to deliver nonpharmacologic interventions that are individualized for older adults. Prescribed interactive routines were feasible to use in older adults who self-reported loneliness and pain. Routine engagement may be influenced by the older adults’ pain characteristics and personalities. Tailoring these routines to align with individual pain profiles and personality traits may improve the efficacy of interventions and user satisfaction. A personalized approach can enhance adherence and overall outcomes by addressing specific needs and preferences of older adults, making technology-assisted interventions more relevant and efficient in managing their conditions.

## Supplementary material

10.2196/64763 Checklist 1 CONSORT guidelines for randomized pilot study.

## Abbreviations

AI: artificial intelligence
BFI: Big Five Inventory
CVA-E: conversational voice assistant enhanced
CVA-S: conversational voice assistant standard
SUS: System Usability Scale
